# Commentaries on Diseases of the Skin

**Published:** 1840-07

**Authors:** 


Art. XI.—*Commentaries on Diseases of the Skin, illustrated by coloured Plates, representing the Commencement, Progress, and Termination of the Eruptions, drawn, lithographed, and coloured by* Joseph Perry. By A. T. Thomson, m.d., f.l.s.., Professor of Materia Medica in University College, London. Parts I-II-III.—*London,* 1839-40.

This is a work of considerable pretension both on the score of its letter-press and its pictorial illustrations; and we are happy in being able to say, after a careful inspection of both, that the pretension is well justified by the performances both of the writer and the artist. The plates are, indeed, excellent, both as to design and colouring, and fulfil almost every requisite that illustrations are capable of supplying. We can therefore, strongly recommend the work to our readers, well assured that the closest study of it will justify our recommendation.

The only fault we have to find with the publication is its price; although we by no means think it dear, when we consider the quality of its contents; on the contrary, we think it very cheap; but we mean to express our fears that its price may prevent it from coming into the hands of practitioners generally, and the great body of the profession be thereby deprived of much valuable instruction. We trust, at any rate, that every medical society, library, and book-club, will take care that its members have this advantage.

The three fasciculi published contain eight folio plates and about 150 pages of octavo letter-press; the subjects treated of and represented being Lepra vulgaris, L. syphilitica; Psoriasis diffusa, P. palmaria et lotorum, P. inveterata; Rupia vulgaris; Pityriasis rubra; Herpes zona.

The author's account of the different diseases is very full yet practical; the treatment is given at considerable length; and numerous cases are introduced both from the private and hospital practice of Dr. Thomson. The style is rather careless; and the improper expression "the habit,"—meaning body, system, constitution, temperament, &c. &c.—provokes the reader in every page.

